# An Actor-Based Model of Social Network Influence on Adolescent Body Size, Screen Time, and Playing Sports

**DOI:** 10.1371/journal.pone.0039795

**Published:** 2012-06-29

**Authors:** David A. Shoham, Liping Tong, Peter J. Lamberson, Amy H. Auchincloss, Jun Zhang, Lara Dugas, Jay S. Kaufman, Richard S. Cooper, Amy Luke

**Affiliations:** 1 Department of Preventive Medicine & Epidemiology, Loyola University Chicago, Maywood, Illinois, United States of America; 2 Department of Mathematics & Statistics, Loyola University Chicago, Chicago, Illinois, United States of America; 3 Kellogg Graduate School of Management, Northwestern University, Evanston, Illinois, United States of America; 4 Department of Epidemiology & Biostatistics, Drexel University, Philadelphia, Pennsylvania, United States of America; 5 Department of Epidemiology, Biostatistics & Occupational Health, McGill University, Montreal, Quebec, Canada; University of Washington, United States of America

## Abstract

Recent studies suggest that obesity may be “contagious” between individuals in social networks. Social contagion (influence), however, may not be identifiable using traditional statistical approaches because they cannot distinguish contagion from homophily (the propensity for individuals to select friends who are similar to themselves) or from shared environmental influences. In this paper, we apply the stochastic actor-based model (SABM) framework developed by Snijders and colleagues to data on adolescent body mass index (BMI), screen time, and playing active sports. Our primary hypothesis was that social influences on adolescent body size and related behaviors are independent of friend selection. Employing the SABM, we simultaneously modeled network dynamics (friendship selection based on homophily and structural characteristics of the network) and social influence. We focused on the 2 largest schools in the National Longitudinal Study of Adolescent Health (Add Health) and held the school environment constant by examining the 2 school networks separately (N = 624 and 1151). Results show support in both schools for homophily on BMI, but also for social influence on BMI. There was no evidence of homophily on screen time in either school, while only one of the schools showed homophily on playing active sports. There was, however, evidence of social influence on screen time in one of the schools, and playing active sports in both schools. These results suggest that both homophily and social influence are important in understanding patterns of adolescent obesity. Intervention efforts should take into consideration peers’ influence on one another, rather than treating “high risk” adolescents in isolation.

## Introduction

Childhood obesity is epidemic in the U.S. [1], [2]. Recent data show that that 18.1% of adolescents (ages 12–19 years old) are obese (defined as exceeding the historical 95^th^ percentile of age- and sex-specific body mass index (BMI)) [3]. By contrast, the prevalence of U.S. adolescent obesity in the period 1988–1994 was 10.7% [4]. To reverse the alarming rise in childhood and adolescent obesitỳ, researchers have tried many individual-level prevention strategies, including educating children on healthy eating habits, promoting increased physical activitỳ, and restricting screen time. Most interventions, however, have shown, at most, modest benefit. For example, a 2011 Cochrane Review by Waters and colleagues showed that interventions aimed at reducing obesity in 13-to-18 year old adolescents lowered BMI by an average of 0.09 kg/m^2^ [5]. The failure of these interventions, especially those targeting individuals, has spurred researchers to identify social and economic influences and suggest novel population-level interventions [6]. Along these lines, recent studies support an etiologic role for social networks in the production and maintenance of childhood and adult obesity [7], [8], [9], [10].

Social relationships and interactions generally influence behaviors and health outcomes [11], [12]. We may represent the pattern of relationships between “social entities” as a social network; the entities might be individuals, collectives (such as households), institutions, or governments [13]. Social networks are increasingly regarded as important determinants of health issues as diverse as the spread of human immunodeficiency virus [14] and the “contagion” of several conditions including obesity [7], smoking [15], [16], [17], and even happiness [18]. Valente has further shown the importance of social networks in the diffusion of health-related innovations and behaviors [19]. The specific mechanisms by which networks influence behavior are poorly understood, although norms [11], [20], peer modeling [21], and social capital [22] have all been implicated.

Notable critiques of the social network “contagion” hypothesis have appeared in academic [23], [24], [25], [26] and popular [27] literatures. The key issues highlighted by these critiques are a trio of potential mechanisms that could account for the observed “network contagion”: 1) social influence; 2) confounding by shared social environments of network members; and 3) social selection or homophily (“love of sameness”) on shared predisposition to engage in (un)healthy behaviors. These mechanisms are not identifiable using traditional statistical approaches. This trio has been a longstanding problem in epidemiology and other fields, and is best articulated by Manski as the “reflection problem”, because all three mechanisms can mirror one another [28]. The critique is most sharply articulated by Shalizi and Thomas [26]. Using graphical causal models, they show that those aspects of latent traits that lead to *either* friendship formation *or* behavior “must be made observable… In either case the confounding arcs go away, and the direct effect [of peer influence] becomes identifiable” ([26], p.218).

Several prior studies have employed regression-based approaches to adolescent obesity or BMI using data drawn from the National Longitudinal Study of Adolescent Health [24], [25], [29]. These studies all claim to control for confounding by holding constant individual background characteristics that influence behavior. Cohen-Cole and Fletcher offer perhaps the best example of a regression-based approach [24] as their model adds controls for environmental confounding using school-specific trends; these controls alone attenuate the association by over 30%. They further extend Christakis and Fowler’s model by examining the *change* in BMI following declaration of friendship using individual fixed effects (FE). The FE model is appealing because it automatically adjusts for all time-invariant background characteristics of individuals, whether or not these characteristics are observed.

The stochastic actor-based model (SABM) of Snijders and colleagues provides a means of separating the effects of social influence and friend selection [30], [31]. The SABM simultaneously models the evolution of social network structure and the behavior of individuals in the network. In this paper, we apply the stochastic actor-based framework to data on adolescent body size and obesity-related behavior. Our primary hypothesis was that social influences on adolescent body size and obesity-related behavior are independent of peer selection when stratified by the school environment. We predicted that peers exert an influence on one another’s BMI, screen time, and playing active sports; these influences are assumed to be localized in the social network and were operationalized as assimilation (i.e., individual becoming more similar to their friends).

## Methods

The Loyola University Chicago Institutional Review Board approved these analyses. All subject data were de-identified prior to receipt of the data by the investigators, and the study was deemed “exempt”.

### Study Population

Data were drawn from the first and second waves of the National Longitudinal Study of Adolescent Health (hereafter, Add Health). Details of the overall study design, including codebooks, may be found elsewhere [32]. Add Health invited all students at 16 schools to participate in a detailed survey conducted in the student’s home. Only 2 schools enrolled enough students to permit school-stratified analyses and thus only 2 schools are included in the current study, referred to as “Jefferson High” by Bearman [33] and “Sunshine High” by Moody (unpublished data). Jefferson High, located in the rural Midwest, is the only public high school in the area, which is critical because friendships can only be identified if they are within the school. Jefferson High is primarily comprised of non-Hispanic white students. Sunshine High is in an urban environment and has substantial racial and ethnic diversity; this makes it an ideal contrast to the more homogeneous population of Jefferson High.

The total student participation rates were 776 (75.8%) at Jefferson High and 1744 (82.9%) at Sunshine High. Wave 1 was collected during the 1994–1995 school year a follow-up visit took place 1 year later (Wave 2). Because we are interested in longitudinal changes in the social network, we excluded any respondent not followed in Wave 2, which, for the most part, included those who were 12^th^ graders in Wave 1. This yielded a final dataset of 624 students in Jefferson High and 1151 students in Sunshine High for analysis. The remaining schools in the saturation sample (i.e., those not included in this study) only included from 19 to 133 students with complete BMI information; they were not included because of their low sample sizes which would have precluded disentangling peer influence from social selection. To rule out unmeasured confounders at the school level, we stratified all analyses by school.

### Obesity-related Measures

Body size was assessed by BMI (in kg per meters squared); both weight and height were self-reported, as Wave 1 lacked objective measures of these variables. Although self-reported weight was found to be under-reported in Wave 2 of Add Health, the amount was less than 1 pound for males and less than 2 pounds for females [34]. Over one year of followup, there was little transition between adiposity categories using CDC sex- and age-specific BMI cutpoints at the 85^th^ (overweight) and 95^th^ (obese) percentiles [35]. Because only 42 respondents (6.7%) from Jefferson High and 84 (7.4%) from Sunshine High moved up one or more weight categories, we chose to analyze one-unit changes in BMI as the behavioral outcome. As our modeling approach required behaviors to be ordered categories, we recoded BMI as an integer.

Since BMI is a proxy for adiposity rather than a behavior *per se*, we selected two behaviors for investigation that have been implicated in childhood obesity: screen time and (not) playing active sports [36]. Screen time was assessed as the sum total of hours watching television and/or video recordings plus computer or video games in the past week. Implausible values (i.e., above 99 hours per week; n = 4 in Jefferson High and n = 2 in Sunshine) were re-coded as 99 hours. To aid estimation and interpretation, screen time was divided into 10-hour categories ranging from 0 (under 10 hours of screen time) to 9 (90 or more hours per week). Playing active sports was measured with the question: “During the past week, how many times did you play an active sport, such as baseball, softball, basketball, soccer, swimming, or football?” The active sports score was coded as 0 (not at all), 1 (1 or 2 times), 2 (3 or 4 times), or 3 (5 or more times).

### Social Network Measures

At both waves 1 and 2, all respondents were asked to name up to 10 friends, up to 5 male and 5 female. Based on these answers, an N by N *adjacency matrix* for each high school was created, where N is the number of students in the network. If student *i* named student *j* as a friend, then the *i,j* entry in the matrix was a one, and all other entries were zero [13]. Thus, each row of the matrix corresponds to a particular student *i,* called an “ego,” and each ego is surrounded by his or her local “alters”: other actors in the network with their own attributes, network properties, and behaviors, indexed by the subscript *j*, corresponding to the columns in the adjacency matrix (these and other key terms used throughout the paper are defined in **Table 1**). At baseline (Wave 1), further questions assessed the strength of each named friend; however, this information was not used in the present analysis. Only respondents with network (friendship) data were included in the analysis, as only they may serve as both egos and alters.

**Table 1:** Key terms used in this paper.

Term	Definition
Actor	a respondent in one of the Add Health saturation schools
SABM	stochastic actor-based model
Ego	the actor whose network and behavior choices are being modeled
Alter	an actor who is named as a friend by the ego
Degree	the total number of alters an ego has named
Reciprocated tie	tie for which the alter also names the ego as a friend; synonymous with mutual tie
Transitive triplets	triplet whereby one of the ego’s alters names a second of the ego’s alters as a friend; “friend of a friend” who is named by the ego as a friend
Identical attribute	indicates that both the ego and the alter have the same attribute value; a measure of homophily for discrete attributes (sex, grade, and race-ethnicity)
Similar attribute	the standardized absolute difference between the ego’s and the alter’s attribute; a raw (uncentered) value of 1 indicates perfect similarity; used as a measure of homophily for continuous attributes and behaviors
Average similarity	the value of similar behaviors, averaged across all of the ego’s alters; average similarity is used as a measure of peer influence or assimilation
Peer influence	the effect of alters’ behavior on ego’s behavior
Social influences	synonym for peer influence

### Stochastic Actor-based Model (SABM) of Peer Selection and Social Influence

Snijders and colleagues have developed an stochastic actor-based model of the co-evolution of social networks and behaviors [30], [31], implemented in R as the Simulation Investigation for Empirical Network Analysis (R-SIENA). The model uses rate functions to assign type of change (network or behavior) for each individual (actor). Two discrete choice functions are fitted recursively: one for network choices (i.e., friendship selection and dissolution), and one for changes in behavior (in our case, BMI, screen time, or playing active sports). The outcome is a log-linked objective function of the various actors and network attributes, which can be likened to the utility of a particular action for each actor. Actors are more likely to choose actions that yield larger objective function values. But since the model is stochastic, actors may choose lower values as well (albeit with lower probability).

The model parameters are estimated using method-of-moments [37]. The initial network, behaviors, and attributes are used as the starting point of the model, which is then simulated for a given set of parameters, with the results compared to the observed data. The parameters are then adjusted and the model is re-simulated in an iterative cycle to minimize the difference between simulation and observation for all actors based on target statistics for those attributes. Standard errors are calculated using a score function method as described in the R-SIENA manual [38].

### Specification of the SABM Model

Although numerous network and behavior statistics can be included in the model [30], [31]. We included only those statistics that theory or prior research suggested would contribute to a critical network or behavior change. Specifically, we defined **X** to be the friendship adjacency matrix described above.

For the network model, the complete objective function for network state *x* for actor *i* given covariates *y* and behavior *z* is defined as:


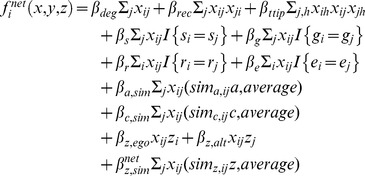


where *deg* indexes degree (number of ties between ego *i* and alters *j*), *rec* indexes reciprocity, *ttip* indexes transitive triplets, *s* is sex, *g* is grade, *r* is black race, *e* is Hispanic ethnicity, *a* is age, *c* is income, and *z* is the behavior variable in question (BMI, screen time per week, or playing active sports score). The variable *x_ij_* is a dummy variable coded 1 if ego *i* names alter *j* as a friend, and 0 otherwise; *x_ij_x_ji_* is coded 1 if *i* and *j* are mutual friends (i.e., it is a reciprocated tie). Likewise, *x_ih_x_ij_x_jh_* is coded 1 if ego *i* and alter *j* both name another person *h* as a friend, and 0 otherwise.

The linear combination of all terms results in a value for *f_i_^net^*(*x,y,z*), the objective function for actor *i*. We may convert this value into a probability for a particular action, exponentiating it, and then dividing by the sum of all possible exponentiated actions. Because the network objective function is complex in its entirety, we describe each of its components below. Each component of the model carries a parameter estimate (*β*), interpreted as the weight the actor places on a particular characteristic of his or her network ties. We divide these into three categories: structural effects, homophily effects, and behavior effects on the network.

### Structural Effects

1. **Outdegree** is defined by the formula *β_deg_*Σ*_j_x_ij_*, where Σ*_j_x_ij_* is the total number of named friends, and *β_deg_* is the parameter (weight placed on adding, keeping, or dropping a new alter), regardless of that alter’s characteristics. Because social actors cannot sustain an unlimited number of friendship ties, *β_deg_* is always negative.
2. **Reciprocity** is the effect of the ego naming a friend if the alter has named the ego as a friend, and is defined by the formula *β_rec_*Σ*_j_x_ij_x_ji_.* Since *x_ij_x_ji_* only takes the value 1 if both ego *i* and alter *j* name each other as friends, Σ*_j_x_ij_x_ji_* is the total sum of mutual ties.
3. **Transitive triplets** is defined as the effect of the ego *i* naming alter *j*’s friend *h* (friend of a friend). The formula is *β_ttip_*Σ*_j,h_x_ih_x_ij_x_jh_*, where *x_ih_x_ij_x_jh_* takes the value 1 if actor *i* names actor *h*, actor *i* names actor *j*, and actor *i* also names actor *h*. Thus, the sum over *j* and *h* is the total number of actors to whom *i* is tied who are also friends with each other.

### Homophily Effects for Actor Attributes

1. **Same sex** is the effect of the number of ties the ego has with alters of the same sex, defined as *β_s_*Σ*_j_x_ij_I*{*s_i_ = s_j_*}, where *I*{*s_i_ = s_j_*} takes the value 1 if both *i* and *j* are the same sex.
2. **Same grade** (*β_g_*Σ*_j_x_ij_I*{*g_i_ = g_j_*}), **same black race** (*β_r_*Σ*_i_x_ij_I*{*r_i_ = r_j_*}), and **same Hispanic ethnicity** (*β_e_*Σ*_i_x_ij_I*{*e_i_ = e_j_*}) are defined analogously to same sex. Because of its racial and ethnic homogeneity, same race and same Hispanic ethnicity are omitted from the model for Jefferson High.
3. 8–9. **Age similarity** and **income similarity** quantify how much weight actors place on choosing friends of similar age and income. They are calculated using the sum of similarity scores between the ego and his or her alters for age, *β_a,sim_*Σ*_j_x_ij_*(*sim_a,ij_ – sim_a,average_*), and for income, *β_c,sim_*Σ*_j_x_ij_*(*sim_c,ij_ – sim_c,average_*). We defined similarity for age (*a*) between ego *i* and alter *j* to be *sim_a,ij_ = *1– [|*a_i_ – a_j_*|/(*a_range_*)], where *a_range_* is the difference between the largest and smallest value of age in the network. The measure for each dyad is centered by subtracting the mean similarity, *sim_a,avg_*, from the similarity measure for that dyad. Income similarity is calculated by substituting income for age in this formula. Since household income was missing for many respondents (17% in Jefferson, and 39% in Sunshine), we substituted the mean value for the school for these actors.

### Behavior Effects on Network

1. **Behavior ego** is interpreted as extra activity or *sociability* for egos with high values of the behavior (BMI, screen time, or active sports). It is calculated as *x_i+_z_i_*_,_ the outdegree weighted by the value of the behavior.
2. **Behavior alter** is interpreted as the *attraction* of the ego to alters with high values of the behavior. It is calculated as the sum of the behavioral value over all of the ego’s alters, Σ*_j_x_ij_z_j_*. When the parameter estimate for the behavior alter effect is negative, this indicates a preference to establish or maintain friendships with alters with low values of the behavior.
3. **Behavior similarity** is the statistic for homophily on behavior. It is calculated as the centered sum of similarity scores between the actor and all of his or her alters, Σ*_j_x_ij_*(*sim_z,ij_ – sim_z,avg_*), using the same general formula employed for age and income similarity. Actors are assumed to prefer alters who are most similar to themselves with regard to behavior (BMI, screen time, and active sports).

### Behavior Objective Function

For the behavior model, the complete objective function for network state *x* for actor *i* given covariates *y* and behavior *z* is defined as:
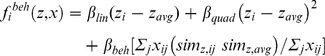
There are three parameters for the behavioral model: linear and quadratic “shape” parameters and the average similarity effect.

1. **linear shape** effect (*z_i_-z_avg_*) and **quadratic shape** (*z_i_-z_avg_*)*^2^* effects are both centered by subtracting the mean value of the behavior (*z_avg_*). The *linear shape* parameter (*β_lin_*) may be likened to the “tracking” of a behavior over time. Subjects who are already higher than average on the behavior are likely to increase it, while subjects who are lower are less likely to do so. The *quadratic shape* effect allows for non-linearity in this association, whereby extreme values at one time point may lead to even more extreme values at a future time point. Snijders and colleagues argue that a positive and significant value for the quadratic shape parameter *β_quad_* indicates addictive behavior [30].
2. **Behavior average similarity** is defined as Σ*_j_x_ij_*(*sim_z,ij_ – sim_z,avg_*)/Σ*_j_x_ij_*. The focus of our analysis is on this effect, as it represents behavioral social influence or assimilation. If the parameter *β_beh_* makes a meaningful contribution to the behavior objective function, then it indicates that egos whose behavior differs from that of their peers assimilate to their peers by increasing or decreasing the behavior. With BMI, this may indicate a conscious decision to lose weight in order to fit in with lean friends, or an unconscious choice of unhealthy foods based on imitating peer behavior.

Note that SIENA requires separate models for each investigated behavior. To rule out unmeasured confounders at the school level, and since schools define the boundaries of the social networks, we stratified all analyses by school. Because there are two schools (Jefferson and Sunshine) and three behaviors examined (BMI, screen time, and playing active sports), a total of 6 models were run.

## Results

### Descriptive Statistics

The characteristics of students in the two schools are listed in **Table 2**. Respondents in each school were similar on age, percent male, and playing active sports. Average household income was $11,500 higher in Jefferson High than Sunshine. Both BMI (1.7 kg/m^2^) and screen time (3.5 hours/week) were higher in Sunshine High than Jefferson and Jefferson High respondents reported more friendships (3.5 vs. 1.8 per student) resulting in a higher overall number of ties (2201 vs. 2025), despite fewer students. There were also a greater number of average reciprocated ties (mutual friendships) and transitive triplets (the friend of an alter’s friend is also the ego’s friend) in Jefferson compared to Sunshine. The similarity measures are centered by the overall average in the network, and thus are close to zero.

**Table 2:** Respondent characteristics at baseline (Wave 1), unless otherwise noted.

	Jefferson High	Sunshine High
Number of respondents	624	1151
Age	16.1 (1.1)	16.5 (0.9)
Range of grades	9–11	10–11
Male	47.4%	49.9%
Non-Hispanic Black	0.0%	21.3%
Hispanic	0.8%	40.6%
Household Income ($1 k)	45.2 (26.7)	33.7 (18.8)
Mean BMI (kg/m^2^, raw)	21.9 (4.4)	23.6 (4.7)
Mean BMI (integer, both time points)	22.6	23.3
Range of BMI (min – max)	13.8–44.3	15.5–51.4
Screen time (h/wk)	14.9 (14.7)	18.6 (15.3)
Active sport score	1.5 (1.1)	1.3 (1.1)
Total number of ties	2201	2025
Out-degree	3.5 (2.3)	1.8 (1.8)
Reciprocated ties	1.4 (1.4)	0.59 (0.94)
Transitive triplets	2.6 (4.1)	0.86 (2.18)
Sum of BMI similarities^1^	0.095 (0.357)	0.041 (0.179)
BMI avg. similarity^2^	0.017 (0.092)	0.015 (0.068)
Sum of screen time similarities	0.060 (0.325)	−0.006 (0.244)
Screen time avg. similarity	0.015 (0.090)	−0.005 (0.104)
Sum of active sport similarities	0.111 (0.449)	0.054 (0.330)
Active sport avg. similarity	0.029 (0.129)	0.021 (0.145)

For continuous measures, mean values are given with standard deviations in parentheses. For categorical variables, percentages are given.

1 “Sum of BMI similarities” is the mean value for the *total sum of* BMI similarities between the actor and each of his or her alters.

2 “BMI average similarity” is the mean value for the *average* similarity between an actor and his or her alters.

### Network Objective Function

Parameters for the network objective function that were common to all models were robust to the inclusion of different behavioral attributes; that is, network structural parameters (degree, reciprocity, transitive triplets, and homophily on sex, grade, black race, Hispanic ethnicity, age, and income) are not confounded by behavioral attributes of actors, and did not change appreciably across models. **Table 3** shows network structural characteristics:. all but one of the parameter estimates in this table make meaningful contributions to the objective function for adding or deleting a network tie: income similarity in Jefferson High which was close to zero with a wide confidence interval. The estimates may be likened to the weight that each individual places upon network and alter attributes in deciding to add or drop a friendship tie or to keep his or her personal network as it is. Out-degree is strongly negative, reflecting the disinclination to form ties with random alters. Reciprocity, however, is strongly positive, indicating that an ego is highly inclined to form or keep friendship ties with alters who have named the ego as a friend. The values for sex, grade, black race, Hispanic ethnicity, age, and income quantify the weight placed on homophily for these attributes.

**Table 3:** Structural influences on network for Jefferson and Sunshine High, parameters and (95% confidence intervals).^1^

	Jefferson High	Sunshine High
basic rate parameterfriendship^2^	12.87	6.77
1: outdegree (density)^3^	−3.56 (−3.64, −3.48)	−5.97 (−6.21, −5.73)
2: reciprocity^4^	2.26 (2.13, 2.39)	2.48 (2.31, 2.66)
3: transitive triplets^5^	0.48 (0.43, 0.53)	0.67 (0.59, 0.75)
4: same sex^6^	0.18 (0.10, 0.26)	0.47 (0.37, 0.57)
5: same grade^6^	0.49 (0.41, 0.57)	0.51 (0.40, 0.61)
6: same black race^6^		0.83 (0.71, 0.95)
7: same Hispanic ethnicity^6^		0.91 (0.74, 1.08)
8: age similarity^6^	0.91 (0.62, 1.20)	1.18 (0.80, 1.56)
9: income similarity^6^	0.060 (−0.23, 0.35)	0.56 (0.21, 0.90)

1 Parameters are the weights actors place on various network configurations. They are the contributions to the objective function. The 95% confidence intervals quantify the precision of the estimates a score function method.

2 The basic rate parameter for friendship controls how often actors have the opportunity to change their network (add, keep, or drop a friend). Higher values indicate more network changes.

3 The outdegree parameter is the weight placed on having a friendship tie with *any* member of the social network, irrespective of the alter’s characteristics.

4 The reciprocity parameter is the weight an actor places on reciprocating alters’ friendship nominations.

5 The transitive triplets parameter is the weight an actor places on naming friends who are also named by the actor’s friend.

6 Positive values of “same” and “similarity” measures are the effects of homophily on these attributes.

Parameters for the network objective function change across models when we examine different behaviors (**Table 4**). These differences arise from each behavior having its own distribution, and actors giving the behaviors different weights. The *attractiveness* measures represent the weight egos place on alters’ behavior; positive measures indicate that egos prefer alters who are above average on the behavior, while negative measures indicate a preference for those below the mean on those behaviors. Positive *sociability* (called “activity” by Snijders et al. [30]) measures indicate that egos are more likely to form ties if they have above-average values of the behavior. Finally, *similarity* measures indicate a preference for alters who have values that are similar to the ego’s values on the behavioral attribute. In both Jefferson High and Sunshine High, we found evidence of homophily on BMI, with a parameter estimate of 0.54 and 95% confidence interval (0.14, 0.95) for Jefferson, and 1.30 (0.68, 1.91) for Sunshine. In both schools, high BMI students chose friends who were similarly high in BMI, while lean students chose lean friends. Ego’s BMI also made a small contribution to sociability; all things being equal, students with high BMI named more friends than those who are low on BMI. Sensitivity analyses, including additional controls for screen time similarity and playing active sports similarity, did not meaningfully change these results.

**Table 4:** Behavioral influence on network choice for Jefferson and Sunshine High, parameters and (95% confidence intervals).

	Jefferson High	Sunshine High
**BMI models**		
10: Attractiveness of alters who are high on BMI^1^	−0.007 (−0.017, 0.003)	−0.009 (−0.021, 0.003)
11: Ego’s BMI (sociability)^2^	0.014 (0.003, 0.030)	0.017 (0.003, 0.031)
12: Similarity of ego’s and alter’s BMI^3^	0.54 (0.14, 0.95)	1.30 (0.68, 1.91)
**Screen time models**		
10: Attractiveness of alters with high screen time	−0.017 (−0.104, 0.069)	−0.043 (−0.142, 0.056)
11: Ego’s screen time (sociability)	0.023 (−0.071, 0.117)	−0.066 (−0.169, 0.037)
12: Similarity of ego’s and alter’s screen time	0.17 (−0.94, 1.28)	−0.89 (−2.25, 0.47)
**Playing active sports models**		
10: Attractiveness of alters playing active sports more often	0.082 (0.019, 0.144)	0.061 (−0.022, 0.143)
11: Ego’s playing more active sports (sociability)	0.021 (−0.05, 0.091)	−0.061 (−0.148, 0.026)
12: Similarity of ego’s and alter’s active sports frequency	0.59 (0.21, 0.96)	0.28 (−0.20, 0.76)

Network change parameters are adjusted for structural (Table 3) and behavior change parameters (Table 5).

1 Positive values for attractiveness indicate that egos generally prefer to become or maintain friendships with alters who have high levels of the BMI or behavior; negative values indicate a disinclination to keep or maintain friendships with individuals with high levels of the BMI or behavior.

2 Sociability indicates that egos with high levels of BMI or the behavior prefer to have more friends.

3 Similarity is the measure of homophily on BMI or the behavior. Positive values indicate a preference for alters whose values are similar to the ego’s.

Jefferson High showed evidence of homophily on active sports. Respondents who reported playing active sports more often were more likely to choose others who also played more often, perhaps because they chose friends who played the same sports. We note that when all forms of physical activity (active sports, exercising, and rollerblading or bike riding) were combined to create a summary score, neither school showed evidence of homophily (results not shown). Playing active sports, however, did not appear to be a basis for friendship selection in Sunshine High; this may have been due to the lower density of that network. Against our prediction, screen time did not appear to affect the actors’ choice of friends in either school.

To illustrate how the network objective function is calculated, consider a respondent from Jefferson High who is male, 17 years old, in grade 11, and with a BMI of 25 (we do not include income similarity or attractiveness of alter’s BMI here because the parameter estimates make ignorable contributions to the objective function). The student has 2 friends, one of whom reciprocates, and one who does not; the alters are male, both in grade 11, and both with a BMI of 25. The alters are not friends with each other. The network objective function for this student’s current network is a linear combination of parameters for outdegree (−3.56), reciprocity (2.26), transitive triplets (0.48), identical sex (0.18), same grade (0.49), age similarity (0.91), sociability (0.14), and BMI similarity (0.54). Similarity scores are calculated as described above, yielding the following formula:
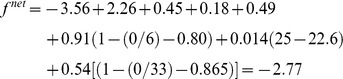
Suppose this student is contemplating dropping his male friend who has not reciprocated, or adding a third male friend who is obese (i.e., has a BMI of 30), but who has named the ego as a friend, thus creating a reciprocated friendship tie. This third male student is also 17 and in grade 11. We may calculate the predicted probability of dropping, adding, or keeping the same ties for any individual in the network by exponentiating the value of the objective function for a particular scenario, and dividing it by the sum of the exponentiated objective values for all possible scenarios. If our network contained only the four individuals described here, the ego could make four possible choices: keep the same network; drop one of the 2 existing ties; or add the tie that is not present. The exponentiated values of these four choices, and the probability of each, would be:

1. Keep network the same: exp(−2.77) = 0.0624; p = 0.0624/0.9584 = 0.065
2. Drop tie with reciprocating alter: exp(−2.52) = 0.0807; p = 0.084
3. Drop tie with the non-reciprocating alter: exp(−0.26) = 0.7737; p = 0.807
4. Add tie to alter with BMI of 30: exp(−3.18) = 0.0415; p = 0.043

Note that the denominator for each probability (p) is 0.9584, the sum of the four exponentiated objective function values for each choice (0.0624+0.0807+0.7737+0.0415). In this artificial scenario, it is most likely the student will make the third choice, that is, to drop the existing unreciprocated tie. This choice has the highest probability because the parameters are obtained from a school containing 624 individuals, but we are applying them to a hypothetical network of only 4 individuals which is high in density (0.25, as there are 3 ties over 4×3 or 12 possible ties). In reality, the network is already low in density (density is 0.006, as only 2201 of the 624×623 possible ties are present). There are 624 network choices possible for a student at Jefferson High, or as many choices as there are actors in the network, and the value of the objective function for each choice would need to be calculated and compared to estimate the predicted probability of any particular choice.

### Behavior Objective Function

Values for the behavior objective function parameters are listed in **Table 5**. We found evidence of peer influence (social modeling or assimilation) for BMI and playing active sports in both Jefferson and Sunshine High, and for screen time in Jefferson High.

**Table 5:** Network influence on behavior, parameters and (95% confidence intervals).

	Jefferson High	Sunshine High
Rate parameter for BMI behavior	4.17	5.20
13: BMI linear shape	0.16 (0.11, 0.22)	0.10 (0.06, 0.13)
14: BMI quadratic shape	0.015 (0.004, 0.025)	0.006 (−0.0003, 0.012)
15: BMI average similarity	14.10 (7.76, 20.44)	10.57 (5.30, 15.85)
Rate parameter for screen time behavior	3.97	7.39
13: Screen time linear shape	−0.46 (−0.59, −0.34)	−0.36 (−0.426, −0.293)
14: Screen time quadratic shape	0.070 (0.013, 0.126)	0.012 (−0.008, 0.032)
15: Screen time average similarity	5.04 (0.07, 10.00)	−0.47 (−2.41, 1.47)
Rate parameter for active sports behavior	3.84	3.77
13: Active sports linear shape	−0.20 (−0.28, −0.11)	−0.33 (−0.40, −0.27)
14: Active sports quadratic shape	0.33 (0.24, 0.41)	0.23 (0.15, 0.30)
15: Active sports average similarity	1.74 (0.66, 2.82)	1.30 (0.27, 2.32)

Behavioral change parameters are adjusted for network structural parameters (Table 3 and 4).

Linear and quadratic shape parameters are the effects of the ego’s own behavior (linear) and behavior-squared (quadratic) on his or her future behavior. The “average similarity” parameters represent social influence of the alters’ on the ego.

### Evidence of Peer Influence on BMI

The BMI average similarity score for Jefferson High was 14.10 (95% CI: 7.76, 20.44). This indicates a tendency for egos to try to match the average BMI of their friends; if their BMIs are higher than their friends, this will tend to pull their BMI down; if they are lower than their friends, it will pull their BMI up. While this parameter estimate seems high, it must be viewed in the context of the mean value (0.017), minimum (−0.54), maximum (0.14), and interquartile range (−0.002 to 0.078) of average similarity values. Thus, at the 25^th^ percentile value, the contribution of average similarity to the objective function is (14.10)(−0.002) = −0.028; at the 75 percentile, it is (14.10)(0.078) = 1.10. The BMI average similarity value for Sunshine High was similar, at 10.57 (95% CI: 5.30, 15.85). Sensitivity analyses, including additional controls to the behavior objective function for sex, ethnicity, race, age, income, body weight image, trying to lose weight, and trying to gain weight, did not meaningfully change these parameter estimates (results not shown).

The behavior objective function is simpler than the network function because there are only three choices that an actor can make: stay the same; move up one unit; or move down one unit. The larger the value is of the objective function, the greater the probability of the choice made, and it will depend both on the ego’s BMI and the average similarity with his or her alters. Ego’s current BMI influences future BMI, as indicated by the “linear shape” and “quadratic shape” parameters. As current BMI values increase, there is a greater tendency to increase BMI between time steps; that is, more emphasis is placed upon increasing BMI than decreasing it. Translating the behavior objective function into probabilities is done in an analogous fashion to the calculation for network changes. We exponentiate the value of the objective function for a particular BMI state, and then divide it by the sum of the exponentiated objective function values for all three scenarios (move down one unit, stay same, or move up one unit).

To illustrate, consider a student in Jefferson High whose BMI is 23, close to the mean value (22.6). We can assume the student is male; while sex is not a part of the behavior objective function, it *is* a determinant of the student’s friends. If the actor has no friends, then only the linear and quadratic shape will drive the objective function values of his 3 choices:

1. Drop one unit of BMI (i.e., go from 23 to 22): exp(−0.093) = 0.912; prob = 0.278
2. Stay at the same BMI (23): exp(0.0689) = 1.071; prob = 0.327
3. Increase one unit of BMI (from 23 to 24): exp(0.260) = 1.30; prob = 0.395

The most probable scenario is that the actor will increase his BMI by one unit, but the other two scenarios are nearly as likely.

Now consider a situation where this same ego has 2 friends, each with the identical BMI of 30 kg/m^2^. The centered average similarity value between this ego and his friends is thus:
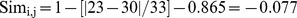
Were he to move up one unit in BMI, the centered similarity measure would become larger (0.047); were he to move down one unit, similarity would become smaller (−0.107). These measures then figure into the objective function, and each “move” in BMI can be assigned a probability:

1. Drop one unit in BMI: exp(−0.093+(14.10)(−0.107)) = exp(−1.608) = 0.200; p = 0.163
2. Stay at the same BMI: exp(−1.019) = 0.361; p = 0.293
3. Increase one unit BMI: exp(−0.402) = 0.669; p = 0.544

It is more likely than not that this subject will increase his BMI. The converse, however, is not true: going down in BMI when alters are lower on BMI is much less likely than gaining body mass when the alters are higher. If the scenario were reversed, with the ego’s BMI beginning at 30 and the alters’ at 23, the probability of decreasing BMI is 0.351, while the probability of increasing BMI is 0.319. **Table 6** shows the probability of increasing, decreasing, or remaining at the same BMI for various combinations of egos’ BMI and average similarity with alters’ BMI. The table shows that egos who have alters with higher BMI will be more likely to be pulled in the alters’ direction, while egos with leaner alters do not necessarily have higher probabilities of moving down.

**Table 6:** Probability of ego’s increasing (+1), decreasing (−1), or remaining at the same body mass index (BMI) in the next time step, based on ego’s and average alters’ current BMI.

		Average current value of alters’ BMI (kg/m^2^)			
Ego’s current BMI (kg/m^2^)	Change	20	25	30	35
**20**	**−1**	0.26	0.18	0.18	0.18
	**same**	0.43	0.30	0.30	0.30
	**+1**	0.31	0.51	0.51	0.51
**25**	**−1**	0.40	0.22	0.15	0.15
	**same**	0.33	0.42	0.29	0.29
	**+1**	0.27	0.35	0.56	0.56
**30**	**−1**	0.35	0.35	0.19	0.12
	**same**	0.33	0.33	0.41	0.27
	**+1**	0.32	0.32	0.40	0.61
**35**	**−1**	0.30	0.30	0.30	0.16
	**same**	0.33	0.33	0.33	0.40
	**+1**	0.37	0.37	0.37	0.45

### Predicted Behavior on Screen Time and Playing Active Sports

Similar calculations can be made for peer influence in Jefferson High on screen time (**Table 7**) and playing an active sport **(** **Table 8**). Results (**Table 7**) show that egos with low values of screen time are unlikely to increase their screen time if their alters are similar in screen time. Egos who are high on screen time are likely to remain high or increase their screen time if their alters spend much time in front of the TV or computer. Because of the negative linear and positive quadratic shape contributions to the objective function, egos in the middle are more likely to be influenced by extremes of peer behavior: egos with a screen time of 30–39 hours per week are most likely to reduce their screen time if their alters are low on screen time (10–19 hours/week) (probability of decrease = 0.61), whereas egos who are medium-high viewers at 60–69 hours/week are most likely to increase time if their average alter watches 80–89 hours/week (probability of increase = 0.62).

**Table 7:** Probability of changing ego’s screen time in the next time step, based on ego’s and average alters’ current screen time score (in 10 hour intervals).

		Average alters’ screen time (10 hour intervals)			
Ego’s current screen time (10 hour intervals)	Change	1	3	6	8
**1**	**−1**	0.41	0.31	0.31	0.31
	**same**	0.42	0.32	0.32	0.32
	**+1**	0.16	0.37	0.37	0.37
**3**	**−1**	0.6	0.33	0.22	0.22
	**same**	0.27	0.45	0.3	0.3
	**+1**	0.14	0.23	0.47	0.47
**6**	**−1**	0.46	0.46	0.21	0.13
	**same**	0.31	0.31	0.44	0.26
	**+1**	0.24	0.24	0.34	0.62
**8**	**−1**	0.36	0.36	0.36	0.15
	**same**	0.32	0.32	0.32	0.42
	**+1**	0.33	0.33	0.33	0.43

Change means increasing or decreasing by one 10 hour interval, or staying at the same level.

A similar pattern is noted for playing active sports (**Table 8**). Egos who played an active sport once or twice in the past week at Wave 1 had a 75% predicted probability of decreasing their playing sports if their average alter did not play any sports. On the other hand, egos who played sports 3–4 times a week at baseline had a 62% probability of increasing their playing sports if their average alter played 5 or more times.

**Table 8:** Probability of changing ego’s playing active sports score in the next time step, based on ego’s and average alters’ current active sports score.

		Average alters’ active sports score			
Ego’s current active sports score	Change	0	1	2	3
**0**	**−1**	*na*	*na*	*na*	*na*
	**same**	0.81	0.57	0.57	0.57
	**+1**	0.19	0.43	0.43	0.43
**1**	**−1**	0.75	0.48	0.36	0.36
	**same**	0.17	0.36	0.27	0.27
	**+1**	0.08	0.16	0.38	0.38
**2**	**−1**	0.55	0.55	0.27	0.16
	**same**	0.24	0.24	0.39	0.23
	**+1**	0.21	0.21	0.34	0.62
**3**	**−1**	0.54	0.54	0.54	0.27
	**same**	0.46	0.46	0.46	0.73
	**+1**	*na*	*na*	*na*	*na*

The playing active sports score is the frequency in the past week: 0 = not at all; 1 = 1 or 2 times; 2 = 3 or 4 times; 3 = 5 or more times. Egos may increase by one level, decrease by one level, or stay at the same level.

## Discussion

Our model’s primary strength is that we explicitly model both the processes of friendship formation and social influence. Our results add to a growing body of evidence demonstrating clustering of friends’ obesity and related behaviors [7], [10], [25], [29]. All of these previous models employ a variation of the generalized estimating equation (GEE), which accounts for the correlated structure of the data, but does not explicitly model social network dynamics. While showing that BMI and behaviors cluster is *consistent* with a causal story that friends influence one another (or that obesity spreads through social networks), GEE models offer little *support* of such a causal claim. The crux of the problem lies in the potential for confounding by shared environments and homophily [26], or the tendency of similar individuals to form friendships as in the adage “birds of a feather flock together” [39]. Controls for shared environments can be introduced using traditional methods, such as adjusting for neighborhood characteristics or including controls for fixed effects, as done by Cohen-Cole and Fletcher [24]. Homophily is more difficult to control for, since it may be based not only on the behavior in question (which is observed), but also on unobserved (latent) tendencies for friendship formation (e.g., a shared propensity for the behavior, which may itself be due to race, sex, or other characteristic). Unless the shared propensity toward both the behavior and the friendship is controlled for, we cannot progress beyond merely documenting a correlation of behavior between friends.

We found that a number of well-known bases for homophily operate in the Add Health friendship network, including sex, age, grade, race-ethnicity, and income [39]. Our findings are also consistent with two works by de la Haye [9], [40] and one by O’Malley [41] that findevidence for homophily on body size using SABM, exponential random graph and tie prediction models. While we found evidence that homophily matters for BMI and for playing active sports, we found no evidence for homophily on screen time. Because the model allows for homophily in friendship retention and in dropping ties, results should be robust to the “unfriending problem” described by Noel and Nyhan [42]. After accounting for these many sources of homophily (age, race-ethnicity, income, sex, and grade), we found evidence of social influence for BMI, screen time, and playing active sports. These results contrast with de la Haye and colleagues’ SABM analysis [40], which did not find any evidence of peer influence on BMI once homophily and other structural factors were taken into account. These differing results may be due to their study’s smaller sample size (N = 156), the Australian setting, or a different specification of the influence parameter (alter’s BMI, rather than similarity between ego’s and alters’ BMI, as in our model).

Our model further extends prior work by specifically examining behaviors implicated in the epidemic of childhood obesity. The model is based on observations of respondents from two large high schools that are quite different, yet the results show substantively similar evidence of peer influence on BMI and playing active sports. Estimates of social influence in the two schools are not directly comparable, because these measures depend upon such factors as the average behavioral values and ranges for the school and the density of network ties. For example, effects for influence may have been smaller in Sunshine High due to the sparseness of its in-school social network, as reflected in the lower average out-degree (1.8, vs. 3.5 in Jefferson High). Differences in the built environment between the two schools may also have contributed to heterogeneity of effects [43]. Because we stratified the analyses to respondents within two schools, the school environment cannot be a confounder. Stratification further allowed us to demonstrate internal validity, as qualitatively similar results for peer influence on BMI and playing active sports were obtained in both schools.

Our model also addresses a major limitation of regression-based approaches. As noted by Salizi and Thomas, peer influence effect can only be identified if the mechanism for friendship formation can be specified, measured, and included in the model [26]. Our model does provide such specification for friendship selection, based on reciprocity, transitivity, and homophily on several characteristics, including the behavior in question (BMI, screen time, and playing active sports). Our modeling framework also captures feedback between selection and influence processes. For example, large weight gain may be stigmatizing and lead to social isolation [44], in which case the beneficial effect of having (leaner) friends would be missed. Alternatively, obese adolescents might form and maintain friendships only with other obese adolescents; if social influence is present, then the two processes would be reinforcing. Regression models, which assume individual observations to be independent, cannot handle this type of complexity.

There are several limitations to our study. First, we rely on self-reported BMI, screen time, and frequency of playing active sports. Self-reported BMI is known to suffer from cross-sectional misclassification bias based on sex, age, and race-ethnicity [45], [46]. However, because sex and race-ethnicity are constant across waves and age only differs by one year, *change* in BMI might be underreported but should not otherwise be biased. Field and colleagues found that while obese males and females underreported weight by the largest margin, weight *change* showed relatively little bias (underreported by 1.7 pounds in males, and over-reported by 0.3 pounds in females) [34]. Likewise, screen time may be underreported and playing sports over-reported, but these biases should be consistent across waves.

A second is our use of observational data. There is no feasible mechanism for randomly assigning friendships, which would be the most satisfying means of removing homophily as a competing explanation, although some forms of dyadic relationship may be assigned. In a “natural experiment” of the random assignment of college freshmen roommates, researchers found that obese women negatively influence weight gain in their roommates, perhaps through eating behavior [47]. Nevertheless, roommates are not necessarily friends, and the results of this study cannot be directly compared to the current results. Another approach would involve the random assignment of obesity status to one node of a dyad. The only “natural experiment” we can identify, however, is a study by Woodard and colleagues of weight loss following a spouse’s bariatric surgery [48]. Because of the observational nature of our data, we lacked some measures that may have confounded the findings of peer influence. In particular, we did not have a measure of Tanner stage. Physical maturation is an important contributor to individual BMI trajectories and physical activity [49], and it is plausible that more developed adolescents were both more likely to be friends and also more likely to increase BMI.

A further limitation is that the SABM model is designed for discrete behaviors, such as smoking and alcohol consumption [30], [31]. On the behavior side, the SABM requires that increases or decreases occur in single unit quanta, and is unable to handle continuous behavioral outcomes. In addition, SABM was designed for small networks (up to a few hundred actors). In small networks, each actor has the opportunity to form ties with all other actors [30], an assumption that is unlikely to hold in our analyses. Running analyses on such large networks was computationally intensive: each model took several hours on an 8-core machine to complete. Finally, SIENA can only model one behavior at a time, precluding simultaneous modeling of peer influence on BMI, screen time, and playing active sports. In future studies, we hope to address some of these limitations by extending the SABM framework to handle continuous behavior measures in large networks with greater computational efficiency. For the present time, however, the implementation in R-SIENA is the only means capable of teasing apart network dynamics and social influence.

In conclusion, we found support for social influence on obesity-related measures and behaviors that is independent of homophily or confounding by shared school environment. Nevertheless, homophily on BMI and playing sports cannot be ignored. We will use these model results to parameterize an agent-based model of peer influence and selection processes. In the absence of direct experimentation (such as the natural experiments described earlier), it remains unclear how social networks can be harnessed to promote health or prevent obesity. Regardless, evidence on the importance of social networks continues to accumulate. For intervention purposes, networks may provide an explanation for why “high-risk” approaches that focus only on obese individuals *qua* individuals are prone to fail [50]. Networks may also offer insight into what Sterman terms “policy resistance, [which] arises when we do not understand the full range of feedbacks surrounding… our decisions” ([51] p.507). Our model shows that social influence tends to operate more in detrimental directions, especially for BMI; a focus on weight loss is therefore less likely to be effective than a primary prevention strategy against weight gain. Effective interventions will be necessary to overcome these barriers, requiring that social networks be considered rather than ignored.
